# Editorial: Application of new approach methodologies in toxicological assessment of next-generation tobacco and nicotine products

**DOI:** 10.3389/ftox.2026.1843194

**Published:** 2026-05-15

**Authors:** Ranulfo Lemus, K. Monica Lee, Rosalia Emma, Massimo Caruso, David M. Wood

**Affiliations:** 1 Letox World LLC, Dayton, OH, United States; 2 Crestview Consultation LLC, Manakin-Sabot, VA, United States; 3 Department of Medicine and Surgery “Kore” University of Enna, Enna, Italy; 4 Department of Biomedical and Biotechnological Sciences, University of Catania, Catania, Italy; 5 Clinical Toxicology, Guy’s and St Thomas’ NHS Foundation Trust, King’s Health Partners and King’s College London, London, United Kingdom

**Keywords:** ENDS (electronic nicotine delivery systems), heated tobacco products (HTPs), NAMs (new approach methodologies), NGPs (next-generation tobacco and nicotine products), OTDN (oral tobacco-derived nicotine), OoC (organ-on-a-chip), AOPs (adverse outcome pathways), ALI (air-liquid-interface)

There is ongoing concern over the use of animals to test the safety of substances and consumer products, including novel tobacco products. While animal testing of tobacco products is prohibited in many countries (e.g., Belgium, Estonia, Germany, Slovakia, and the United Kingdom), the United States regulatory agency (FDA) states, in its 2011 draft guidance on applications for premarket review of new tobacco products, that manufacturers evaluate the toxicity, abuse liability, and carcinogenicity of new products “using some combination of *in vitro*, *in vivo*, and/or *ex vivo* studies.” *In vivo* studies are similarly listed among the types of non-clinical studies described in US FDA, 2025 draft guidance on modified risk tobacco product (MRTP) applications. While there is an increasing interest in the use of exclusively non-animal and *in silico* methods to support marketing applications for new products (US FDA, 2026a; US FDA, 2025), the Family Smoking Prevention and Tobacco Control Act (FSPTCA) states that the FDA’s decision regarding whether a tobacco product may be marketed is ultimately based on “*well-controlled investigations, including nonclinical and clinical data*”, focusing on the protection of public health. In the EU, Directive 2010/63/EU has encouraged the transition towards reducing animal testing and promoting the use of new approach methodologies (NAMs) in line with the principles of replacement, reduction, and refinement (known as the “3R”). However, its practical impact on regulatory data requirements has been uneven across different regulatory contexts (European Parliament resolution, 2010).

Overall, NAMs are intended to replace animal studies due to the ethical concerns of using animals, alongside the challenges in animals providing sufficient clinically relevant risk assessment, due to the necessity for large safety factors and the limited predictive value of animal models in relation to human diseases (Monticello et al., 2017; Clark and Steger-Hartmann, 2018). NAMs offer several advantages, including the use of relevant human cells, investigation of human-specific disease mechanisms, provision of mechanistic insights to improve predictive capabilities, development of microphysiological systems that mimic physiological conditions, and integration with Artificial Intelligence (AI) to enhance analysis and interpretation (Schmeisser et al., 2023).

NAMs, which include *in vitro* and computational (*in silico*) methods, are becoming valuable tools for safety and risk assessment for novel tobacco and nicotine products (Lee et al., 2022; Thorne et al., 2024). Although these tools are growing in use, many are not truly “new”; what is novel is their broader applications beyond traditional hazard identification allowing regulatory decisions based on NAMs without requiring new animal tests. In this Research Topic, we presented six peer-reviewed original studies aimed at advancing NAM approaches which highlight the use of *in vitro* tools for evaluating next-generation tobacco and nicotine products (NGPs), such as heated tobacco products (HTPs), nicotine pouches (NPs), and electronic nicotine delivery systems (ENDS).

Researchers from both academic institutions and the tobacco industry^1^ contributed their testing data and perspectives about the use of selected NAM tools, highlighting strengths, limitations, and potential opportunities in evaluating NGPs. The studies (Cao et al.; Chapman et al.; Hayashida et al.; Ichikawa et al.; Ito et al.; Wong et al.) cover a wide range of novel nicotine and tobacco products, including oral tobacco-derived nicotine (OTDN) products (Cao et al.), HTPs (Chapman et al.; Hayashida et al.), and ENDS (Chapman et al.; Wong et al.). These publications utilized various NAM platforms, such as cell-based assays (Cao et al.; Wong et al.; Ichikawa et al.), organ-on-a-chip systems (Chapman et al.; Hayashida et al.), omics technologies (Wong et al.), adverse outcome pathways (Ichikawa et al.), and *in silico* models (Ito et al.). Below is a summary of the NAMs used in these studies; readers are encouraged to review the six relevant publications for more detail.

Cell-based assays employing human-derived cells and tissues allow investigation of chemical effects within a controlled laboratory setting, enhancing the clinical relevance to toxicity outcomes. Cao et al. compared the biological impact of OTDN products—extracted using artificial saliva and complete artificial saliva—on cytotoxicity, oxidative stress, and inflammatory responses across four *in vitro* oral models. These included monolayer cultures of normal human gingival fibroblasts (NHGFs) and oral epithelial cells (NHOEs), as well as three-dimensional organotypic models (EpiOral™ and EpiOral™ Full Thickness). For inhalable products, Wong et al. conducted an integrated study exposing three-dimensional (3D) human bronchial epithelial tissues (EpiAirway™) at the air-liquid interface (ALI) to aerosols containing aldehydes generated through the thermal decomposition of electronic cigarette (EC) solvents, followed by proteomic analyses. Ichikawa et al. combined an Adverse Outcome Pathway (AOP) framework with an *in vitro* approach, subjecting 3D cultured human bronchial epithelial cells (HBECs) to whole cigarette smoke (WCS).

Organ-on-a-chip (OoC) systems are microphysiological models cultured on microfluidic chips that supply medium and signals through microchannels. Two studies introduced pathogenesis of atherosclerosis models to evaluate ENDS (Chapman et al.) and HTPs (Chapman et al.; Hayashida et al.). Atherogenic endpoints (oxidative stress, monocyte adhesion, ICAM-1 expression, and inflammatory markers) were assessed on a Human Coronary Artery Endothelial Cells (HCAECs)-chip combined with THP-1 monocytes under flow conditions (Chapman et al.). Endothelial barrier impairment, monocyte adhesion, and monocyte migration were measured on HCAECs-chip. Macrophages were exposed to HTP aerosol extracts, and conditioned medium was collected. HCAECs cultured on chips were exposed to these conditioned media to mimic the effects on the vascular system caused by inflammatory responses (Hayashida et al.).

As supplementary to standardized *in vitro* tools, discovery-focused omics technologies, such as genomics, metabolomics, proteomics, and transcriptomics, are useful to uncover underlying molecular signatures related to adverse outcomes and/or disease. These approaches encourage a move toward mechanistic toxicology, emphasizing early molecular changes over traditional late-stage pathological effects. Utilizing proteomics data, researchers performed *in vitro* experiments on BEAS-2B bronchial epithelial cells, assessing reactive oxygen species, mitochondrial activity, and cytoskeletal stability after short-term exposure to two aldehydes (acetaldehyde or methylglyoxal) at levels commonly found in EC aerosols (Wong et al.). The results from this proteomics research were further confirmed by the concentration-response 2D *in vitro* results, providing insight into the mechanisms by which thermal breakdown of EC solvents, propylene glycol and glycerin, may impact biological systems.

Adverse Outcome Pathways (AOPs) outline how toxicants cause adverse effects through key events (KEs) which track the progression toward an adverse outcome (AO). Ichikawa et al. developed an AOP-based *in vitro* method to test disease-related states that can be reproduced by exposing 3D-HBECs (from six different donors) to WCS and observe both acute phase responses (oxidative stress, epidermal growth factor receptor activation, and SP1 activation) and chronic phase responses (intracellular mucus production, goblet cell metaplasia/hyperplasia, and mucus hypersecretion) along the AOP.

*In silico* models employ computational methodologies to simulate biological responses or predict chemical properties using existing datasets. Ito et al. integrated the AOP of mucus hypersecretion, a well-recognized indicator of chronic airway disease as reported by Ichikawa et al., with probabilistic quantitative models based on Bayesian networks. The incorporation of an AOP-based *in vitro-in silico* approach represents a promising tool for evaluating the chronic inhalation effects associated with consumer products and chemicals.

As more individual NAMs platforms were introduced, integrating NAMs (Vuong et al., 2025) as a holistic alternative tool for toxicologists has become more common for evaluating products, ingredients, and mixtures. The examples include approaches such as cell-based assays combined with omics technologies (Wong et al.), cell-based assays integrated with AOP frameworks (Ichikawa et al.), or in silico-AOP-based cell assay methods (Ito et al.).

Several challenges still remain in accepting NAMs for regulatory purposes, including validation, reproducibility, and the need for robust performance standards. Currently, many NAMs are still used primarily at a developmental stage, with limited validation for regulatory acceptance. As noted in recent FDA draft guidance, regulatory confidence in NAM data depends on the reliability of the methodology and on scientifically accepted validation criteria (US FDA, 2026b). Additional considerations include the need for fit-for-purpose methods, the limited availability of models encompassing the breadth of human disease processes, and the recognition that NAMs may not always function as direct one-to-one replacement for traditional 90-day *in vivo* studies (German Federal Institute for Risk Assessment, 2022; Ouedraogo et al., 2025; Schmeisser et al., 2023). Furthermore, many currently implemented NAMs are predominantly focused on cytotoxicity and related molecular pathways, which may not fully capture the effects of pharmacologically active constituents, including nicotine, or other compounds associated with functional physiological responses. This highlights the need for further methodological development to address a broader range of biologically relevant endpoints (Schmeisser et al., 2023; Ouedraogo et al., 2025).

Overall, the papers included in this Research Topic highlight the growing value of NAMs in the toxicological assessment of tobacco and nicotine products.

## Footnotes

1 Papers from the tobacco industry were accepted by the journal at the time of submission (2025) provided that the authors disclosed any conflict of interest related to the work, the journal has since changed its policy and papers from the tobacco industry are no longer considered (https://www.frontiersin.org/journals/toxicology/about#about-scope).
